# Clinical Characteristics and Prognostic Factors of Children With Anti-N-Methyl-D-Aspartate Receptor Encephalitis

**DOI:** 10.3389/fped.2021.605042

**Published:** 2021-04-22

**Authors:** Sai Yang, Liming Yang, Hongmei Liao, Mei Chen, Mei Feng, Shulei Liu, Lihong Tan

**Affiliations:** Department of Neurology, Hunan Children's Hospital, Changsha, China

**Keywords:** anti-N-methyl-D-aspartate receptor encephalitis, pediatrics, neurology, prognosis, treatment, rituximab

## Abstract

**Objective:** Anti-N-methyl-D-aspartate (anti-NMDA) receptor encephalitis is the most common autoimmune encephalitis in pediatric patients. The study aimed to investigate the clinical characteristics and prognostic factors of anti-NMDA receptor encephalitis in children in South China.

**Methods:** This was a retrospective study of children diagnosed with anti-NMDA receptor encephalitis between 01/2014 and 12/2017 at Hunan Children's Hospital. Laboratory, brain magnetic resonance imaging (MRI), and electroencephalography data were collected. The short-term (6-month) outcomes were assessed using the Liverpool score by the same pediatric neurologist. The children were divided into good (scores 4–5) and poor (score <3) clinical outcomes.

**Results:** Among the 51 patients, 21 (41.2%) were male. The most common clinical symptoms were dyskinesia (88.2%), personality change (84.3%), seizure (82.4%), and cognitive disorder (31.4%). Two were transferred to another hospital, 45 (91.8%) received intravenous immunoglobulins, 41 (83.7%) received methylprednisolone, and 8 (16.3%) received plasma exchange. Eight (16.3%) received rituximab for second-line treatment, six after intravenous immunoglobulin and methylprednisolone treatment, and two after plasma exchange therapy failed. Seven were lost to follow-up. The short-term outcome was good in 23 patients. Cognitive disorder [odds ratio (OR): 23.97, 95% confidence interval (CI): 1.12–513.30, *P* = 0.042) and abnormal brain MRI (OR: 14.29, 95% CI: 1.36–150.10, *P* = 0.027] were independently associated with a poor short-term outcome after adjustment for age, GCS, and rituximab use.

**Conclusions:** MRI abnormalities and cognitive disorders are independently associated with poor short-term outcomes in children with anti-NMDA receptor encephalitis. The use of rituximab is not associated with the 6-month outcomes.

## Introduction

N-methyl-D-aspartate (NMDA)-receptor encephalitis is an acute form of encephalitis caused by an autoimmune reaction to the GluN1 subunit of the NMDA neuronal receptor (1, 2). It usually affects women of reproductive age but can occur in men and patients of all ages (2, 3). Although the prevalence and incidence of anti-NMDA receptor encephalitis remain unclear (4), pediatric patients accounted for 37% of the patients in a previous study (5). Anti-NMDA receptor encephalitis is identified more frequently than any specific viral etiology in children with unknown encephalitis (6). In addition, anti-NMDA receptor encephalitis is a leading cause of autoimmune encephalitis (7, 8). Compared with adults, anti-NMDA receptor encephalitis in children is characterized mainly by seizures, dyskinesia, and localized neurological symptoms (5, 9). Recovery is typically slow and erratic and may take >3 years (2). Relapse occurs in 20–25% of the patients (2, 10). The disease is fatal in 4% of the cases (3).

The first-line treatment for anti-NMDA receptor encephalitis is immunotherapy, including methylprednisolone, intravenous immunoglobulins, and plasma exchange. The second-line treatment is immunosuppression, typically with rituximab or cyclophosphamide. Nevertheless, standardized treatments are not well-defined (5, 8, 9). In addition, who should receive second-line treatment and when second-line treatment should be initiated remain unclear, with wide variations among hospitals. Hence, the use rate of second-line immunosuppression is 14–81% (5, 9, 11–14). According to a recent survey of pediatric neurologists, the time of initiating second-line therapy varies widely, from started at diagnosis (0 months) or at 6 months in the absence of improvement to the primary therapy (8).

The reason for the variability in second-line treatment use is the unclear effect reported in previous observational studies (15–17). In a study by Zekeridou et al. (9), although more patients received second-line treatment, the relapse rate (8%) was similar to that reported in other studies in which fewer patients received second-line treatment (12%) (14). On the other hand, Titulaer et al. (5) reported that second-line treatment was a factor associated with good outcomes.

The first case of anti-NMDA receptor encephalitis in China was reported in 2010, followed by many adult and some pediatric cases (18, 19). Still, very few dedicated studies have investigated anti-NMDA receptor encephalitis in pediatric patients or a South China population. Therefore, this study aimed to describe the clinical characteristics of children with anti-NMDA receptor encephalitis in South China and explore the prognostic factors associated with short-term outcomes.

## Methods

### Study Design

This retrospective observational cohort study was approved by the institutional review board of Hunan Children's Hospital, Changsha, China. The need for individual consent was waived by the committee.

### Subjects

The children diagnosed with anti-NMDA receptor encephalitis between January 1, 2014, and December 1, 2017, at the neurology department of Hunan Children's Hospital of China were included. The diagnostic criteria of pediatric anti-NMDA receptor encephalitis were (1) age 0–16 years, (2) with at least one sign or symptom among the following: abnormal behavior (psychiatric symptoms) or cognitive dysfunction, language dysfunction (continuous, uninterrupted mandatory language, language reduction, silence), seizures, dyskinesia or myotonia, abnormal posture, decreased consciousness, autonomic dysfunction or central hypoventilation, and positive for anti-NMDA receptor (GluN1 subunit) IgG antibody [antibody detection included the cerebrospinal fluid (CSF)] (20), and (3) exclusion of other possible causes of encephalitis (1). All children included in this study had positive results for CSF or serum anti-NMDA receptor (GluN1 subunit) IgG antibody.

### Clinical Data Collection

The demographic information, including age, sex, and parents' educational level, was routinely collected by nurses. The clinical symptoms were recorded and categorized by experienced physicians: fever, seizure, consciousness, movement disorder (e.g., orofacial dyskinesias and choreoathetosis), cognitive disorders, personality change, autonomic dysfunction (e.g., ataxia), and upper respiratory tract infections. Consciousness was assessed using the Glasgow Coma Score (GCS) and classified as severe (GCS: 3–8), moderate (GCS: 9–12), and mild (GCS: 13–15) (21). The Wechsler Intelligence Scale for Children-Fourth edition-Chinese version (WISC-IV-Chinese) (22) was used to assess the cognitive capacities of children of 6–16 years of age when they were conscious, able to communicate, and could take a 15-min test. Considering that the children might not be able to complete all the WISC tests, only two index scores representing speech and memory abilities in the WISC, i.e., the Verbal Comprehension Index (VCI) and the Working Memory Index (WMI), were used in the cohort. These two index scores ranged from 40 to 160. Cognitive disorder was defined as having at least one index score <70. The cognitive capacities of children aged <6 years were assessed by physicians asking questions (e.g., what did you eat for breakfast) based on some items of the Gesell Developmental Schedules. If a child exhibited speech difficulties or memory deficits during these conversations, the physician diagnosed the child with a cognitive disorder (23). Personality change assessment was performed according to the Mental Health Law of the People's Republic of China and referred to the diagnostic criteria in ICD-10 (24), i.e., a significant and persistent change in behavioral patterns and interpersonal relationships for at least 2 months. The results of the measurement of the Health of the Nation Outcome Scale for Children and Adolescents were evaluated (25).

**Figure 1:**
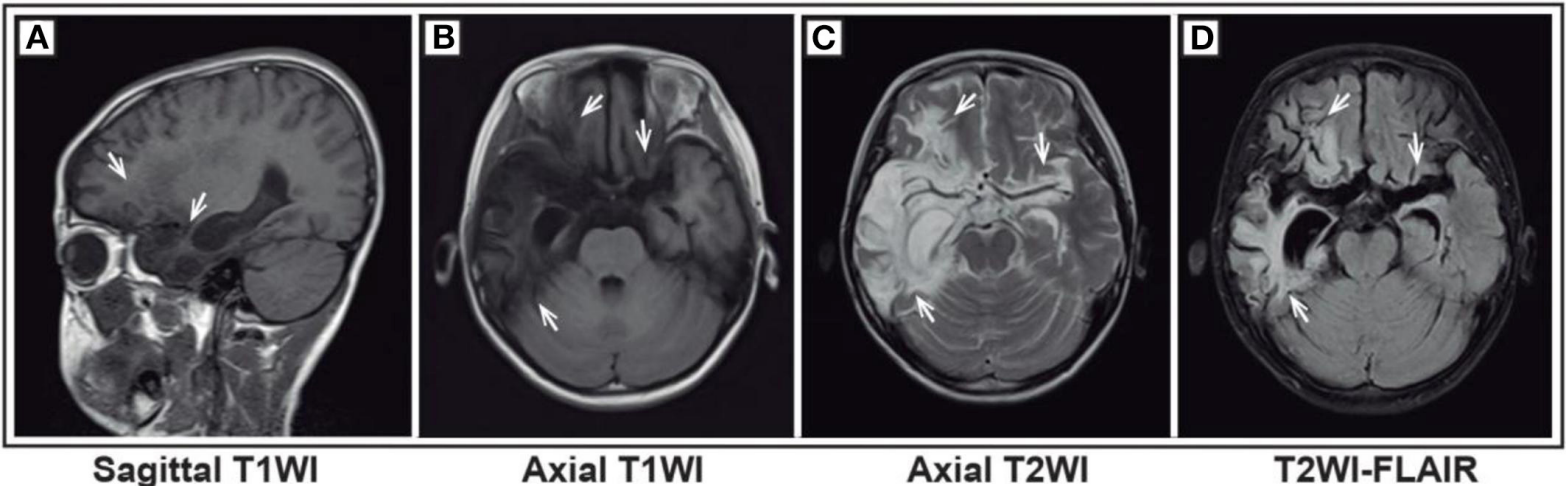
Magnetic resonance imaging (MRI) of a representative pediatric patient admitted for convulsions for 1 week and diagnosed with anti-NMDA receptor encephalitis (NMDA receptor antibody in CSF was 1:10) secondary to HSV encephalitis. The patient was first diagnosed with HSV encephalitis in another hospital. Then, acyclovir was given, but the treatment effect was not good. Bilateral frontal and temporal lobes and insula showed a wide lamellar abnormal signal. The boundary of the lesions was not clear. T1WI showed a low signal **(A,B)**, and T2WI showed a high signal **(C)**. FLAIR sequence was dominated by a high signal **(D)**. The lesion-adjacent brain showed scattered and patchy low signal ditch, crack broadening and deepening, and right ventricle temporal horns. The third ventricle was slightly expanded, with no obvious shift of the midline structure.

**Figure 2:**
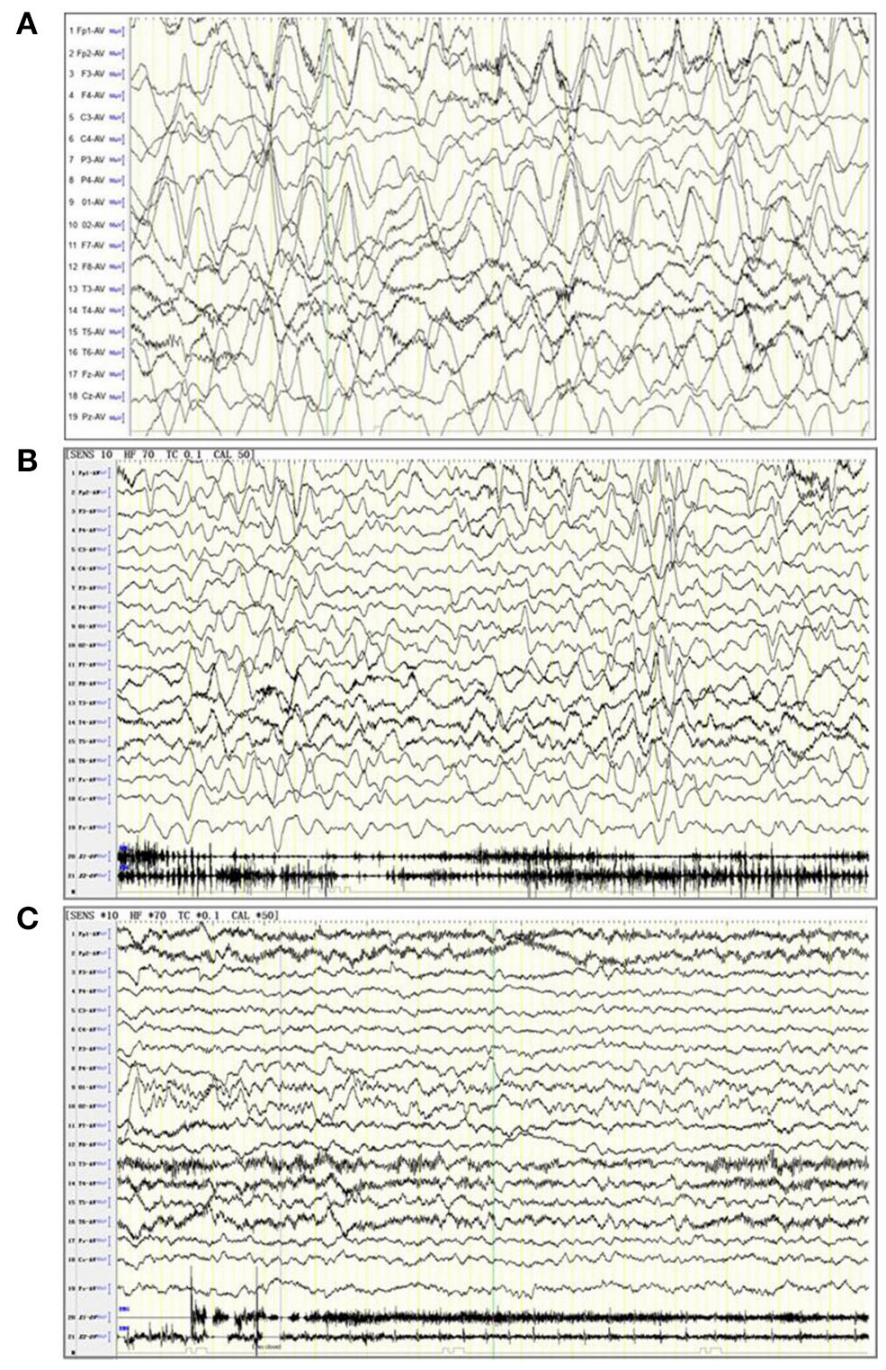
Electroencephalogram (EEG) of three patients. **(A)** A 6-years old female patient with anti-NMDA receptor encephalitis, in a noisy, non-cooperative, awakened state (EEG parameters: SENS: 10 HF, high pass: 70 Tc, low pass: 0.1 Cal, notch value: 50, sampling rate: 1,000, monitoring duration: 2 h). The delta rhythm (1.5–3 Hz, 120–380 UV) was used as the main background, with a small amount of low amplitude beta activity and bilateral symmetry. The background rhythm was significantly moderated. **(B)** A 2 years and 10 months female patient anti-NMDA receptor encephalitis, in an awakened state (EEG parameters: SENS: 10 HF, high pass: 70 Tc, low pass: 0.1 Cal, notch value: 50, sampling rate: 1,000, monitoring duration: 4 h). The delta rhythm (2–3 Hz, 80–300 UV) was used as the main background, with slightly more θ rhythms (4–6 Hz, 50–120 UV) and a small amount of low amplitude beta activity. **(C)** A 5-years old female patient with anti-NMDA receptor encephalitis, in an awakened state (EEG parameters: SENS: 10 HF, high pass: 70 Tc, low pass: 0.1 Cal, notch value: 50, sampling rate: 1,000, monitoring duration: 4 h). The α rhythm (8–9 Hz, 50–90 UV) was the main background in the two occipital regions. The δ and θ waves (2–5 Hz, 40–120 UV) were abundant in each region. The low amplitude β activity was observed in each region, which was symmetrical on both sides. Rhythm regulation and amplitude modulation in the occipital area were poor. The superiority of the occipital area is obvious.

The results from the first magnetic resonance imaging (MRI), electroencephalography (EEG), CSF, and blood after admission were collected. For this study, an abnormal MRI was defined as signs of brain parenchyma damage (any MRI signal abnormality indicating acute injury, infection, or old injury) (Figure 1), and an abnormal EEG was defined as the presence of slow-wave activity and spikes (Figure 2). The laboratory tests for CSF included glucose, protein, and white blood cells (WBC). Herpes simplex virus (HSV) and Epstein-Barr virus (EBV) infection were tested from the blood. Tumor screening was performed for all children. Abdominal and pelvic ultrasound was performed, followed by CSF and serum paraneoplastic antibody testing, including antineuronal nuclear autoantibody 1 (ANNA 1), ANNA 2, and ANNA 3. If teratomas were detected by ultrasound, the patients underwent surgery. If paraneoplastic antibody testing was positive but no teratomas were detected by ultrasound, a chest computed tomography (CT) scan was performed.

### Treatment

The first-line treatment, including methylprednisolone, intravenous immunoglobulins, and plasma exchange (alone or combined), was administered at the time of diagnosis. Second-line rituximab was used if the patients showed no improvements after 7–14 days of first-line treatment (26, 27). No standard treatment protocol regarding dose, time of initiation, and length of each treatment were followed.

### Follow-Up and Short-Term Outcome Assessment

The patients were routinely followed at 6 months after discharge. The children were re-evaluated by the same pediatric neurologist. The Liverpool Outcome Score system was used by the physicians to assess the short-term outcomes of the children at follow-up. The Liverpool Outcome Score system is a commonly used tool to determine a child's level of disability and the likelihood of leading an independent life after Japanese encephalitis (28, 29). It reflects a child's actual neurological status and is specific for children with encephalitis. In addition, based on two field tests in Southeast Asia, including one in India and another in Malaysia, the Liverpool Score System has good inter-observer and intra-observer agreement and good agreement compared with those of clinical assessments (κ = 0.906) (30). The children were divided into two groups. A good clinical outcome was defined as Liverpool Score 5 (full recovery) or 4 (minor sequelae), while a poor clinical outcome was defined as Liverpool Score <3 (moderate or severe sequelae or death).

### Statistical Analysis

SAS 9.4 (SAS Institute Inc., Cary, NY, USA) was used for statistical analysis. Continuous data were tested for normal distribution using the Kolmogorov-Smirnov test. They were presented as means ± standard deviations and analyzed using Student's *t*-test. The categorical variables were presented as numbers (percentages) and analyzed using the chi-square test or Fisher's exact test. The variables associated with the outcome (*P* < 0.10) in the univariable analyses were entered into a multivariable logistic regression analysis. Age is a common prognostic factor and was also included in the multivariable analysis regardless of the univariable analysis results. The results were presented as odds ratios (OR) with the 95% confidence intervals (CI). P < 0.05 (two-sided) was considered significant.

## Results

### Characteristics of the Patients

**Figure 3:**
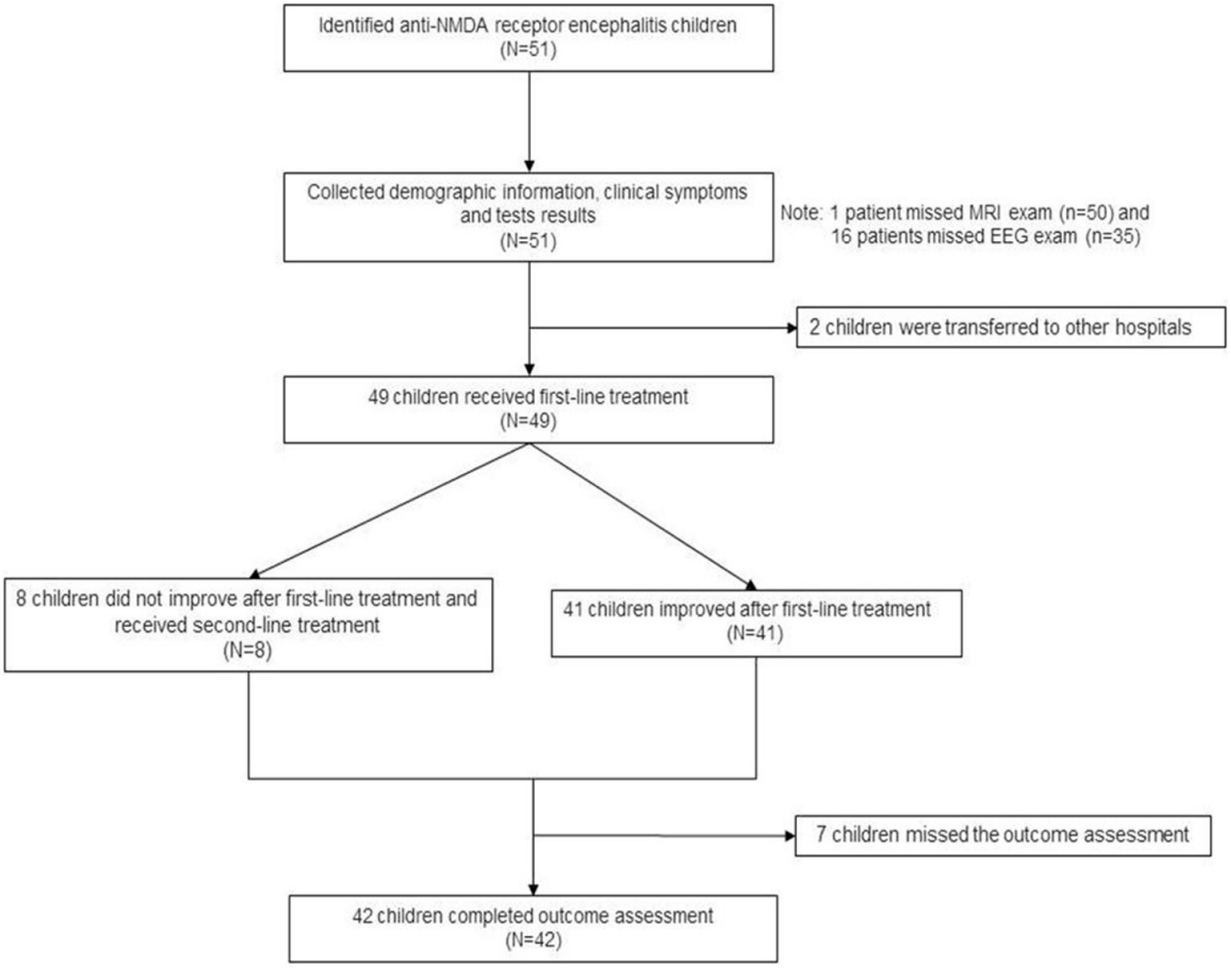
Patient flowchart.

Figure 3 presents the patient flowchart. Of the 51 patients recruited, 2 were transferred to other hospitals after the diagnosis was confirmed, 7 were lost to follow-up, and 42 completed the 6-month follow-up after discharge. Of the 51 patients, 21 were male (41.2%), and 30 were female (58.8%), for a male-to-female ratio of 1:1.42 (Table 1). Mean age was 7.4 ± 3.2 years. There were 16 children younger than 6 years. Among them, 50 were positive for serum anti-NMDA receptor, 48 were positive for CSF anti-NMDA receptor, and 47 were positive for both serum and CSF receptors. The most common clinical symptoms were dyskinesia (*n* = 45, 88.2%), personality change (*n* = 43, 84.3%), epilepsy (*n* = 42, 82.4%), and cognitive impairment (*n* = 16, 31.4%). GCS was >13 points in 33 children (64.7%), 9–12 in 13 (25.5%), and <8 points in five (9.8%). Ten patients (19.6%) had HSV infection and seven (13.7%) had EBV infection. Brain MRI showed brain parenchymal damage in 27 patients (52.9%); among them, the abnormalities were observed in the whole brain (*n* = 1), temporal lobe (*n* = 3), temporal lobe and other parts (*n* = 12), frontal lobe (*n* = 4), frontal lobe, and other parts (*n* = 5), and atypical pathological changes (*n* = 2). EEG revealed slow-wave activity in 42 patients (82.4%). A 12.5-year-old female patient with ovarian teratoma underwent tumor resection.

**Table 1:** Demographics, clinical symptoms, examination results, treatment, and outcome.

**Variables**	**Values (%)**
**Demographics**	
Age (years)	7.4 ± 3.2
**Sex**	
Female	30/51 (58.8%)
Male	21/51 (41.2%)
**Education level of parents**	
Elementary	8/51 (15.7%)
Middle school	15/51 (29.4%)
High school	15/51 (29.4%)
College or higher	13/51 (25.5%)
**Clinical symptoms**	
Seizure	42/51 (82.4%)
Fever	14/51 (27.5%)
Upper respiratory infection	2/51 (3.9%)
Autonomic dysfunction	12/51 (23.5%)
Personality change	43/51 (84.3%)
Movement disorder	45/51 (88.2%)
Cognitive disorder	16/51 (31.4%)
**Glasgow coma scale**	
13–15	33/51 (64.7%)
9–12	13/51 (25.5%)
3–8	5/51 (9.8%)
**Test results**	
WBC in CSF	24/51 (47.1%)
Protein in CSF	32/51 (62.8%)
Glucose in CSF	14/51 (27.5%)
HSV in blood	10/51 (19.6%)
EBV in blood	7/51 (13.7%)
EEG: slow-wave activity	29/35 (82.9%)
EEG: spike	7/35 (20.0%)
**MRI abnormality location**	
Whole brain	1 (2.0%)
Temporal lobe	3 (6.0%)
Temporal lobe and other parts	12 (24.0%)
Frontal lobe	4 (8.0%)
Frontal lobe and other parts	5 (10.0%)
Atypical pathological changes	2 (4.0%)
**Treatment**	
Immunoglobulin	45/49 (91.8%)
Methylprednisolone	41/49 (83.7%)
Plasma exchange	8/49 (16.3%)
Rituximab	8/49 (16.3%)
Outcome in the follow-up (poor)	19/42 (45.2%)

*WBC, white blood cells; CSF, cerebrospinal fluid; HSV, herpes simplex virus; EBV, Epstein-Barr virus; MRI, magnetic resonance imaging; EEG, electroencephalogram*.

Of the 49 patients who received treatment, 45 (91.8%) received intravenous gamma globulin, 41 (83.7%) received methylprednisolone, and 8 (16.3%) received plasma exchange, of whom 4 were severe cases and hence received plasma exchange directly in the intensive care unit. The other 4 patients switched to plasma exchange after intravenous administration of gamma globulin and methylprednisolone. Eight patients (16.3%) were treated with rituximab, 6 of whom received intravenous gamma globulin and methylprednisolone without improvement for 7–14 days and received rituximab directly, and 2 received rituximab after plasma exchange therapy failed. The specific treatment plan was unknown. Supplementary Table 1 presents the treatments of the patients.

### Clinical Factors Influencing the Outcome

The results showed that cognitive disorder (*P* = 0.002) and brain abnormal MRI (*P* = 0.025) were associated with a poor short-term outcome. The use of rituximab (*P* = 0.015) was positively associated with a poor short-term outcome. There was a trend toward children with low GCS scores to have poor short-term outcomes (*P* = 0.051) (Table 2).

**Table 2:** Comparisons of clinical factors between the two groups.

**Variables**	**Poor outcome (*n* = 19)**	**Good outcome (*n* = 23)**	***P***
**Demographics**			
Age (years)	8.0 ± 1.3	6.4 ± 2.1	0.114
Sex			0.976
Female	10 (52.6%)	12 (52.2%)	
Male	9 (47.4%)	11 (47.8%)	
**Education level of the parents**			0.288
Elementary	4 (21.1%)	2 (8.7%)	
Middle school	4 (21.1%)	6 (26.1%)	
High school	8 (42.1%)	6 (26.1%)	
College or higher	3 (15.8%)	9 (39.1%)	
**Clinical symptoms**			
Seizure	15 (79.0%)	21 (91.3%)	0.384
Fever	5 (26.3%)	5 (21.7%)	>0.999
Upper respiratory infection	2 (10.5%)	2 (8.7%)	0.199
Autonomic dysfunction	3 (15.8%)	10 (43.5%)	0.305
Personality change	15 (79.0%)	21 (91.3%)	0.384
Movement disorder	17 (89.5%)	21 (91.3%)	>0.999
Cognitive disorder	11 (57.9%)	3 (13.0%)	0.002
**Glasgow coma scale**			0.051
13–15	9 (47.4%)	19 (82.6%)	
9–12	7 (36.8%)	3 (13.0%)	
3–8	3 (15.8%)	1 (4.4%)	
**Test results**			
WBC in CSF	8 (42.1%)	12 (52.2%)	0.516
Protein in CSF	11 (57.9%)	17 (73.9%)	0.273
Glucose in CSF	3 (15.8%)	7 (30.4%)	0.305
HSV in blood	4 (21.1%)	1 (4.4%)	0.158
EBV in blood	4 (21.1%)	1 (4.4%)	0.158
MRI (abnormal)	14 (73.7%)	9 (39.1%)	0.025
EEG: slow-wave activity	8 (42.1%)	14 (60.9%)	>0.999
EEG: spike	2 (10.5%)	4 (17.4%)	>0.999
**Treatment**			
Immunoglobulin	17 (89.5%)	23 (100.0%)	0.199
Methylprednisolone	15 (79.0%)	21 (91.3%)	0.384
Plasma exchange	6 (31.6%)	8 (34.8%)	0.112
Rituximab	7 (36.8%)	1 (4.4%)	0.015

*WBC, white blood cells; CSF, cerebrospinal fluid; HSV, herpes simplex virus; EBV, Epstein-Barr virus; MRI, magnetic resonance imaging; EEG, electroencephalogram*.

### Multivariable Analysis

In the multivariable logistic regression model, cognitive disorder (OR: 23.97, 95% CI: 1.12–513.30, *P* = 0.042) and abnormal brain MRI (OR: 14.29, 95% CI: 1.36–150.10, *P* = 0.027) were independently associated with a poor short-term outcome after adjustment for age, GCS, and rituximab use. The use of rituximab was not related to the short-term outcomes (*P* = 0.540) (Table 3).

**Table 3:** Association between clinical factors and poor short-term outcome in multivariable analysis.

**Variable**	***P***	**OR**	**95% CI**
Age	0.938	1.02	0.68–1.51
Cognitive disorder	0.042	23.97	1.12–513.30
GCS (9–12 vs. 13–15)	0.556	1.38	0.12–16.04
(3–8 vs. 13–15)	0.212	11.02	0.39-310.65
MRI abnormal	0.027	14.29	1.36–150.10
Rituximab	0.540	2.477	0.14–45.01

*OR, odds ratio; CI, confidence interval; GCS, Glasgow Coma Scale; MRI, magnetic resonance imaging*.

## Discussion

Anti-N-methyl-D-aspartate (anti-NMDA) receptor encephalitis is the most common form of autoimmune encephalitis in pediatric patients. This study aimed to investigate the clinical characteristics and prognostic factors of anti-NMDA receptor encephalitis in children in South China. The results showed that the most common clinical features of pediatric anti-NMDA receptor encephalitis were dyskinesia, personality change, seizure, and cognitive disorders. MRI abnormalities and cognitive disorders were independently associated with poor short-term outcomes in children with anti-NMDA receptor encephalitis. The use of rituximab was not independently associated with the 6-month outcomes.

The most common clinical features in children with anti-NMDA receptor encephalitis were movement disorder, personality change, and seizure. Children with cognitive disorder and brain parenchyma damage demonstrated by MRI were more likely to have a poor short-term outcome. The children more frequently exhibited seizures and movement disorders (both of which >80%) compared with the largest adult case series reported by Titulaer et al. (5). These findings were consistent with Zekeridou et al., who compared 36 pediatric patients and 71 adult patients from the same center (seizure: 50 vs. 23%, movement disorder: 83 vs. 55%) (9). On the other hand, fewer tumors were detected in pediatric patients than in adult patients. Only 1.9% of all pediatric patients (1/51) had tumors in the present study. In contrast, 34% of all the adult patients had tumors in the study by Zekeridou et al. (9), and 52% of all the female patients aged >12 years had tumors in the study by Titulaer et al. (5). The younger the patient, the less likely the tumor was to appear, suggesting that the pathogenesis in children and adults might be different (31).

In 2011, Dalmau et al. (12) pointed out that about 55% of patients with anti-NMDA receptor encephalitis had an abnormal MRI. In 2013, Titulaer et al. (5) reported that 33% of their patients had an abnormal MRI. Zekeridou et al. (9) and Jones et al. (32) reported abnormal MRI in 31% of their patients. Bacchi et al. (33) showed that in children with head MRI abnormalities, the incidence of disturbance of consciousness, probability of recurrence, and Glasgow severity score was higher than in those displaying a normal head MRI. Bartels et al. (34) reported that 39.5% of the children with anti-NMDA receptor encephalitis had abnormal MRI findings, mainly white matter hyperintensities at T2/fluid-attenuated inversion recovery. MRI showed an abnormality in 52.9% of the children in this study, consistent with Dalmau et al. (12). Zekeridou et al. (9) reported that cognitive impairment reached 92%, but cognitive impairment was only 3% in children with prodromal symptoms. Jones et al. (32) reported 56% of cognitive impairment. The present study reported only 31.4% of cognitive impairment, which was significantly lower than in the two studies mentioned above. It was possibly because the children in this study were divided according to <6 and >6 years old, and the WISC-IV and VCI + WMI evaluation systems were adopted to determine the cognitive impairment in the children. The assessment was more complete and detailed. MRI findings in children with anti-NMDA receptor encephalitis showed brain parenchymal damage, which is a factor for poor prognosis. Zekeridou et al. (9) showed that age (>12 years), admission to the ICU, MRI abnormalities, and cognitive impairment were factors for poor prognosis. This was consistent with the findings of the present study. Jones et al. (32) investigated MRI abnormalities associated with poor prognosis, but did not mention cognitive impairment, and only highlighted the level of consciousness. Wang et al. (35) suggested that MRI abnormalities and prognosis were not directly related. Mo et al. (36) showed that age, disturbance of consciousness, and slow waves on EEG were associated with poor prognosis. The clinical characteristics associated with a poor prognosis still need further exploration.

The first-line immunotherapy for anti-NMDA receptor encephalitis includes intravenous gamma globulin, steroids, and plasmapheresis. The second-line immunotherapy includes rituximab and cyclophosphamide (12). For refractory patients, second-line immunotherapy is likely to improve the clinical outcomes (5). Kahn et al. (8) asked 19 questions to 151 pediatric neurologists in 70 different institutions to identify similarities and differences in the treatment of anti-NMDA receptor encephalitis. The start time of the second-line treatment varied greatly, and the effect of the treatment was not clear. The patient's symptoms in the present study did not improve when rituximab was used after 7–14 days of a lack of response to the primary therapy. Only one patient had a good prognosis (1/8, 12.5%). An overall response was seen in seven children (7/8, 87.5%), which was similar to a previous study (5/6, 83.3%) (19). In the univariable analyses, second-line rituximab was associated with the short-term outcome but not in the multivariable analysis. It could be explained by treatment selection bias, a common type of bias in observational studies. In such studies, patients with poor clinical characteristics are more likely to receive second-line treatment and have poor outcomes because of their initial clinical characteristics. The actual treatment effect cannot be estimated without controlling for the treatment selection bias. In this study, the treatment selection bias in a multivariable logistic regression model was adjusted for age, cognitive disorder, abnormal MRI, and GCS, which were variables significantly associated with the outcome in the univariable analyses. After adjustment for the treatment selection bias in the multivariable analysis, rituximab use was no longer significantly related to the outcome.

A significant effect of rituximab on anti-NMDA receptor encephalitis treatment was not observed in this study. Nevertheless, Titulaer et al. (5) reported that second-line treatment resulted in improved outcomes (OR = 2.69). This inconsistency might be due to different follow-up durations; a short-term outcome at 6 months was reported in the present study, whereas the study by Titulaer et al. included an outcome assessment at 4–24 months. In addition, the small sample size of the present study limited the power to detect a significant difference. The results of the present study indicated that brain parenchyma damage shown by MRI and cognitive disorders were predictors of a poor short-term outcome. For patients with abnormal MRI findings and cognitive disorders, the second-line treatment application at the time of diagnosis can be explored rather than waiting until no response is observed from the first-line treatment. However, no standard treatment protocol regarding dose, time of initiation, and length of each treatment were followed. After admission, the patients were first evaluated clinically according to the diagnostic criteria of the Chinese Guidelines for the Diagnosis and Treatment of Autoimmune Encephalitis (2017 Edition). Immunotherapy was initiated in the presence of EEG changes and after excluding other etiologies such as infection, structural changes, etc. Still, in some patients with atypical clinical manifestations, immunotherapy was delayed until the blood and cerebrospinal fluid antibodies were positive. Therefore, there was no uniform timing for the use of immunotherapy among the patients. Studies are necessary to determine the optimal timing of immunotherapy in such patients.

This study has several limitations. The sample size was small, resulting in wide CIs and poor power. In addition, this study was retrospective, and the percentage of missing data was 17.7%, leading to bias. Therefore, the conclusions from this study need to be replicated in a larger sample size. Moreover, the patients were followed for only 6 months, and hence the relapse information and long-term outcomes could not be assessed. Because of different cognitive functions and neurodevelopment, different assessment tools had to be used in children <6 years of age (*n* = 16) and >6 years of age, probably leading to bias. Finally, as per the routine practice at this hospital, the outcomes were only evaluated using the Liverpool score. Future studies could use other tools used in previous studies of anti-NMDA receptor encephalitis, e.g., the NEOS score (37). The use of such tools could allow a more robust and quantitative assessment of the disease outcomes.

In conclusion, the most common clinical features of pediatric anti-NMDA receptor encephalitis are dyskinesia, personality change, seizure, and cognitive disorders. MRI abnormalities and cognitive disorders are independently associated with poor outcomes at 6 months in children with anti-NMDA receptor encephalitis. The use of rituximab is not independently associated with 6-month outcomes. Additional studies are needed to determine the clinical characteristics and prognosis of pediatric anti-NMDA receptor encephalitis.

## Data Availability Statement

The raw data supporting the conclusions of this article will be made available by the authors, without undue reservation.

## Ethics Statement

The studies involving human participants were reviewed and approved by Hunan Children's Hospital. Written informed consent from the participants' legal guardian/next of kin was not required to participate in this study in accordance with the national legislation and the institutional requirements.

## Author Contributions

SY analyzed and interpreted data regarding anti-NMDA receptor encephalitis. SY and MF performed the statistical analysis. MC participated in data collection and statistical analysis. SY wrote the manuscript. All authors read and approved the final manuscript.

## Conflict of Interest

The authors declare that the research was conducted in the absence of any commercial or financial relationships that could be construed as a potential conflict of interest.
