# Supplementary material for: Clinical Characteristics and Prognostic Factors of Children With Anti-N-Methyl-D-Aspartate Receptor Encephalitis
Source: Front Pediatr. 2021 Apr 22;9:605042. doi: 10.3389/fped.2021.605042 (PMC8100243; doi:10.3389/fped.2021.605042)
Supplement: Supplementary file 1 [file Table_1.docx]

Supplementary Material

# Supplementary Tables

**Supplementary Table 1.** Treatment and prognosis of patients (n=49)

| # | Second-line treatment | | | | | Prognosis | | |
| --- | --- | --- | --- | --- | --- | --- | --- | --- |
|  | Methylprednisone 20 mg/kg/d for 3 days | Oral prednisone 2 mg/kg/d | Plasma exchange | IVIG 400 mg/kg/d for 5 days | Rituximab 375 mg/m^2^ 1 week interval | Recovery | Disability | Relapse |
| 1 | √ | √ |  | √ |  | √ |  |  |
| 2 | √ | √ | + | - | Four times |  | √ |  |
| 3 | √ | √ |  | √ |  | √ |  |  |
| 4 | √ | √ |  | √ |  |  | √ |  |
| 5 | √ | √ | + | - | Two times |  | √ |  |
| 6 | √ | √ | + | - |  | √ |  | √ |
| 7 | √ | √ |  | √ |  |  | √ | √ |
| 8 | √ | √ |  | √ | Four times |  | √ | √ |
| 9 | √ | √ | + | - |  | √ |  |  |
| 10 | √ | √ | + | √ |  |  | √ |  |
| 11 |  |  |  | √ |  | √ |  |  |
| 12 | √ | √ |  | √ |  |  | √ | √ |
| 13 |  |  |  | √ |  |  | √ |  |
| 14 | √ | √ | + | √ |  |  | √ |  |
| 15 | √ | √ |  | √ | Four times | √ |  |  |
| 16 | √ | √ |  | √ |  | √ |  |  |
| 17 |  |  |  | √ |  | √ |  |  |
| 18 | √ | √ |  | √ |  | √ |  |  |
| 19 | √ | √ |  | √ | Four times |  | √ |  |
| 20 | √ | √ | + | √ |  | √ |  |  |
| 21 | √ | √ |  | √ |  | √ |  |  |
| 22 |  |  |  | √ |  |  | √ |  |
| 23 | √ | √ | + | √ |  | √ |  |  |
| 24 | √ | √ |  | √ | Four times |  | √ | √ |
| 25 | √ | √ |  | √ | Four times | √ |  |  |
| 26 | √ | √ |  | √ |  |  | √ |  |
| 27 | √ | √ |  | √ | Four times |  | √ |  |
| 28 |  |  |  | √ |  | √ |  |  |
| 29 | √ | √ |  | √ |  |  | √ |  |
| 30 |  |  |  | √ |  | √ |  |  |
| 31 | √ | √ |  | √ |  |  | √ |  |
| 32 |  |  |  | √ |  |  | √ |  |
| 33 | √ | √ |  | √ |  | √ |  |  |
| 34 | √ | √ |  | √ |  | √ |  |  |
| 35 |  |  |  | √ |  |  | √ |  |
| 36 | √ | √ |  | √ |  | √ |  |  |
| 37 | √ | √ |  | √ |  | √ |  |  |
| 38 | √ | √ |  | √ |  | √ |  |  |
| 39 | √ | √ |  | √ |  | √ |  |  |
| 40 | √ | √ |  | √ |  |  | √ |  |
| 41 | √ | √ |  | √ |  | √ |  |  |
| 42 | √ | √ |  | √ |  | √ |  |  |
| 43 | √ | √ |  | √ |  | Miss |  |  |
| 44 | √ | √ |  | √ |  | Miss |  |  |
| 45 | √ | √ |  | √ |  | Miss |  |  |
| 46 | √ | √ |  | √ |  | Miss |  |  |
| 47 | √ | √ |  | √ |  | Miss |  |  |
| 48 | √ | √ |  | √ |  | Miss |  |  |
| 49 | √ | √ |  | √ |  | Miss |  |  |
